# Novel SZT2 mutations in three patients with developmental and epileptic encephalopathies

**DOI:** 10.1002/mgg3.926

**Published:** 2019-08-08

**Authors:** Xiaomin Sun, Xuefei Zhong, Tingsong Li

**Affiliations:** ^1^ Ministry of Education Key Laboratory of Child Development and Disorders Children’s Hospital of Chongqing Medical University Chongqing China; ^2^ Key Laboratory of Pediatrics in Chongqing Chongqing China; ^3^ Chongqing International Science and Technology Cooperation Center for Child Development and Disorders Chongqing China

**Keywords:** compound heterozygous mutations, developmental and epileptic encephalopathy, seizure threshold 2 gene, sequencing, whole‐exome

## Abstract

**Background:**

The seizure threshold 2 (SZT2) gene encodes a large, highly conserved protein that lowers seizure threshold and may also enhance epileptogenesis. In this study, three patients diagnosed with SZT2‐related developmental and epileptic encephalopathies (DEEs) were reviewed aiming to expand knowledge of the genotype and phenotype of SZT2 mutations.

**Methods:**

Targeted next‐generation sequencing was performed to identify pathogenic mutations in 205 cases with DEEs of unknown etiology. Detailed clinical and genetic data were collected from SZT2‐associated patients.

**Results:**

Four novel mutations were found (c.1626 + 1G>A, c.5772dupA, c.4209C > A, c.7307_7308insG) in three patients. All the variants were inherited from their parents. Two patients were siblings and harbored the same mutations and presented developmental delay prior to the onset of seizures. All the individuals were diagnosed as DEEs, drug refractory epilepsy, and experienced status epilepticus (SE); one patient died of SE. One subject showed subependymal nodules as similar as those of tuberous sclerosis complex (TSC) in cranial magnetic resonance imaging (MRI).

**Conclusion:**

Our results expand the genotype and phenotypes of SZT2‐related DEEs, suggesting that SZT2 mutations play a role in developmental delay and epileptic encephalopathy, with high susceptibility to SE and relatively specific MRI findings.

## 1 INTRODUCTION

The developmental and epileptic encephalopathies (DEEs) refer to conditions where epilepsy and neurodevelopmental delay coexist, both of which are supposed to be involved in the pathogenesis (Scheffer et al., 2017).The etiology of epileptic encephalopathy is largely heterogeneous and gene mutations are reported to account for 32% of cases of early‐onset epileptic encephalopathies (Zhang et al., 2017).

The seizure threshold 2 (SZT2, OMIM 615,463) gene, containing 71 exons and located on chromosome 1p34.2, is expressed in the central nervous system and is associated with the regulation of mammalian target of rapamycin (Peng, Yin, & Li, 2017). Biallelic mutations of SZT2 have been shown to reduce the seizure threshold and enhance epileptogenesis in animal studies (Frankel, Yang, Mahaffey, Beyer, & O'Brien, 2009). SZT2 mutation‐related cases have also been reported, presenting mainly with heterogeneous phenotypes ranging from mild‐moderate intellectual disabilities (ID) without seizures (Falcone et al., 2013), to early‐onset epileptic encephalopathy with severe ID (Pizzino et al., 2018). Despite the reports of more than 10 cases (Domingues et al., 2019; Pizzino et al., 2018) of epileptic encephalopathies harboring SZT2 mutations, the clinical features due to SZT2 mutations remain to be elucidated. Here, we report three cases carrying two heterozygous compound mutations and summarize the clinical features, with the aim of expanding our knowledge of the phenotypes and genotypes of SZT2‐related DEEs.

## 2 METHODS

### 2.1 Ethical compliance

All patients provided written informed consent to participation in this study, which was approved by the Medical Ethics Committee of the Children’s Hospital, Chongqing Medical University, China.

### 2.2 Patients

In total, 205 cases diagnosed with unexplained epileptic encephalopathies and DEEs were enrolled in this study from November 2014 to September 2018. All the patients had no obvious etiology of structure, immunology, central nervous system infection, or metabolic disorders according to the clinical manifestation, cranial imaging, and blood/urine screening tests (amino acids and lactate in blood; amino acids and organic acids in urine). All the subjects presented seizure onset and/or developmental delay within the first year after birth. Clinical features, neuroimaging, and electroencephalogram details of patients carrying SZT2 mutations were collected and followed up to February 2019. Mutations in other genes listed in Table S1 were ignored because these were not relevant to the aim of this study.

Genomic DNA was extracted from peripheral blood leukocytes obtained from the enrolled patients and their parents using standard procedures with the QIAamp DNA Blood Mini kits (Qiagen, Germany) according to the manufacturer’s instructions.

### 2.3 Genetic analysis

A gene panel was designed for targeted sequencing of 535 genes (Table S1) that are potentially associated with epilepsy, including SZT2, as described previously (Li et al., 2018). The final DNA libraries were sequenced using the Illumina HiSeq2500 DNA Sequencer to generate paired‐end (90 bp) reads and providing an average coverage depth for each sample of ≥100‐fold. Raw read data were aligned to the reference human genome (UCSC hg19). Single‐nucleotide polymorphisms (SNPs) and indels were then identified. The variants were functionally annotated using an in‐house pipeline as well as the reported frequencies available from public databases (dbSNP 144, HapMap, ESP6500, ExAC, 1,000 Genome Project variants database, and a local control database). For all variants, the results were filtered using a quality value of single base sequencing ≥20. The variants were filtered to identify potential mutation candidates of the following types: (a) functional variants (insertion/deletion: in the coding DNA sequences and splicing region, SNP: nonsense, splice‐site, and missense) and (b) variants with an allele frequency below 0.01 in any of the public databases listed. Known pathogenic variants were identified based on mutations previously reported to cause epilepsy in the literature. The ACMG standards and reference to the NCBI, ClinVar, and HGMD databases were used to classify the mutations into five categories: pathogenic, likely pathogenic, variants of uncertain significance, likely benign, or benign(Amendola et al., 2016). Following the application of the filtering process, the parental origin of the candidate variants was validated by Sanger sequencing.

## 3 RESULTS

### 3.1 Genetic identification of SZT2 mutations

**Figure 1:**
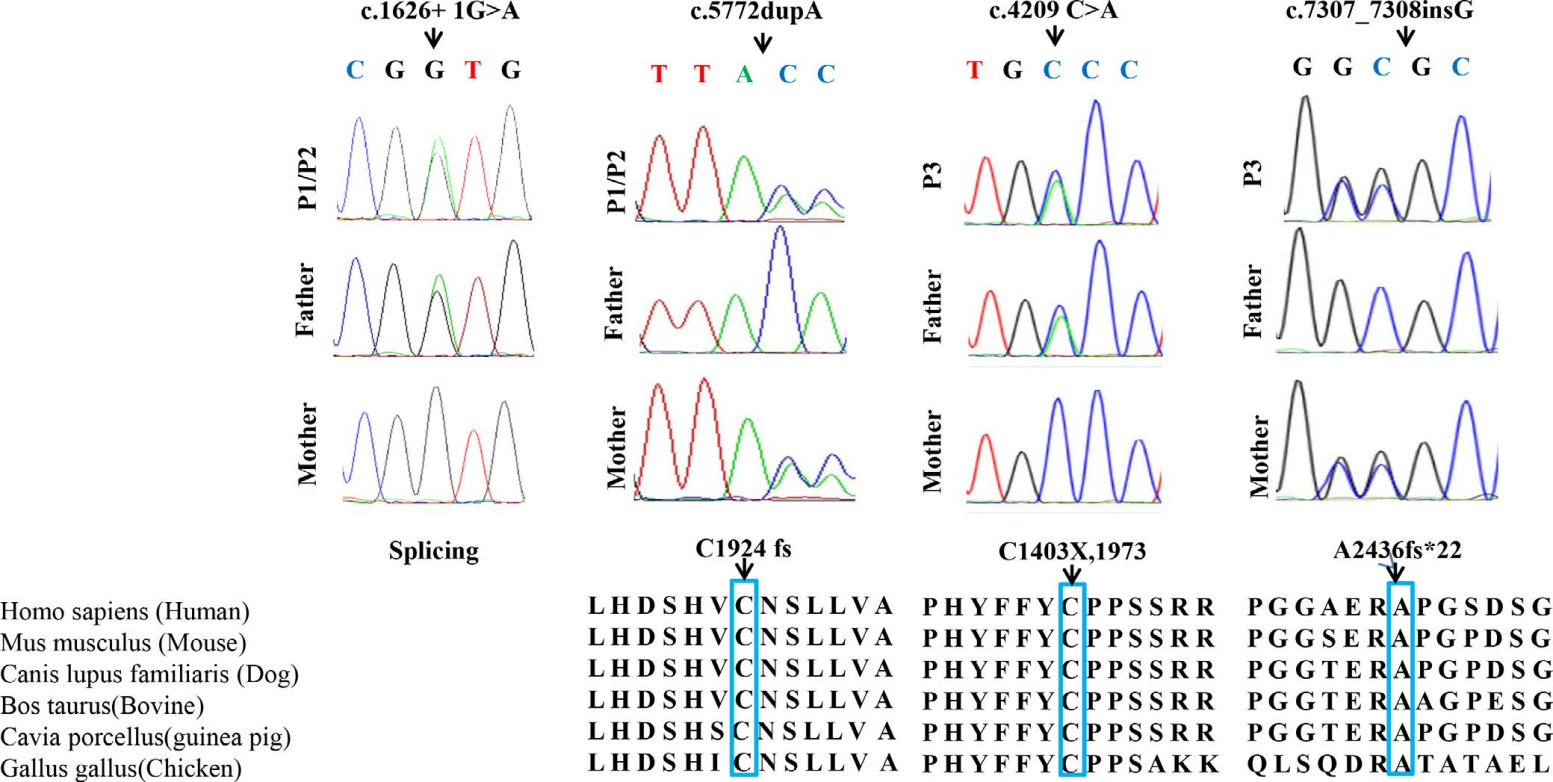
Biallelic SZT2 mutations in three families with epileptic encephalopathy. Sanger sequencing chromatograms confirming the four SZT2 mutations in the identified subjects and their unaffected parents. Positions compared with the NCBI reference gene sequence (NM_015284.3). Conservation of the altered amino acid is shown in the ClustalW alignments

Three patients with compound heterozygous pathogenic SZT2 (NM_015284.3) mutations were identified: c.5772dupA (p.C1924 fs) and c.1626 + 1G>A (splicing) in Patient 1 and Patient 2, and c.7307_7308insG (p. A2436fs*22) and c.4209C > A (p. C1403X, 1973) in Patient 3. Patients 1 and 2 were siblings and harbored the same mutations. All four mutations were novel and inherited from their parents. Details of the SZT2 mutations are shown in Figure 1. We identified one nonsense mutation, two frameshift mutations, and one splice mutation, all of which were predicted to result in protein truncation.

Moreover, the residue of amino acids caused by other three mutations, except c.1626 + 1G>A (splicing), was highly conserved in all vertebrate linages (Figure 1). According to the ACMG guidelines, all four mutations were classified as pathogenic with PVS1 (very strong evidence of pathogenicity 1), +PM2 (moderate evidence of pathogenicity 2), +PM3 in individuals with these variants.

### 3.2 Phenotypes of SZT2 mutations

The clinical characteristics of the three patients are summarized in Table 1. All three cases demonstrated severe developmental delay and refractory seizures.

**Table 1:** Clinical characteristics of patients harboring heterozygous SZT2 mutations

	P1	P2	P3
Sex/age at study	M/ 10 months	F/ 1 year 6 months	M/ 1 year 5 months
Consanguinity	–	–	–
Events in delivery	Cesarean delivery due to maternal pregnancy‐induced hypertension.	Cesarean delivery because of premature placental abruption	Cesarean delivery because of premature placental abruption
Birth weight (g)/Gestation (weeks)	3150/38	2350/36^+1^	2000/31^+6^
Family history	+		−
SZT2 mutations	Compound heterozygous		Compound heterozygous
Paternal allele	c.1626 + 1G>A: splicing		c.4209C > A: p. C1403X,1973
Maternal allele	c.5772dupA: p.C1924 fs		c.7307_7308insG: p. A2436fs*22
Development prior to Sz onset/age at onset of stagnation or regression	Delayed/stagnation at the age of 4 month	Delayed/stagnation at the age of 5 month	Normal/stagnation at the age of 5 month
Sz onset age	10 months	1 year 4 month	4 month
Initial Sz semiology	Cyanosis, eye deviation, and focal tonic and clonic seizures	Twitching of cheek on one side, sialorrhea with or without tonic Sz	Cyanosis, eye deviation, tonic and clonic Sz
Late Sz type	Focal tonic or clonic Sz; SE	Focal or generalized tonic seizures, SE	Focal or generalized tonic Sz, SE
Appearance	High forehead, flattened nasal bridge, hypertelorism	High forehead, flattened nasal bridge, hypertelorism	High forehead
Neurological findings	Hypotonia	Hypotonia	Hypotonia
EEG at onset (age)	hypermaturity of background rhythm (7 months)	Occipital predominance not obvious (11 month)	Normal (4 months 29 days)
EEG at follow‐up	Slow background, sharp‐slow wave complexes in the R frontal area, ictal epileptic discharges without definite origin (10 month 14 days); Slow background; epileptic discharges originating from L medial temporal area on ictal EEG (10 month 27 days);	Slow background; multi‐focal discharges on interictal EEG; epileptic discharges originating from L temporal area on ictal EEG (1 year 6 months)	Slow background with predominance on L parietal; sharp‐slow wave complexes in the L fronto‐parieto‐occiptal areas (5 months); Slow background; multi‐focal discharges with partial generalization (10 month).
MRI (age)	Low T1 and high T2 signals in the white matter aged (6 month 25 days); Enlarged ventricle and delayed myelination in the terminal zone (1 y and 3 month)	Subependymal cyst and widened cavum septum pellucidum (14 days after birth); Normal (11 months)	Cystic signals and widened cavum septum pellucidum (20 days after birth); Subependymal nodules; Shortened corpus callosum; Enlarged ventricles; Widened cavum septum pellucidum (5 month 5 days)
Response to initial treatment	Partial response to LEV	No response to LEV	No response to LEV
Clinical diagnosis	DEE	DEE	DEE
Current treatment and Sz status	Refractory epilepsy; LEV, PB, and OXC; frequent Szs daily	Refractory epilepsy; VPA, NZP, and TPM; Daily or weekly Szs and predisposition to SE	Refractory epilepsy; LEV, TPM, CZP, and VPA; Daily or weekly Szs and predisposition to SE
Clinical examination at the end of follow‐up	Died of SE at 2 year 3 month. Severe DD	2 year 1 month. Unable to crawl, no speech, able to sit independently, hypotonia	1 year 9 month. Unable to raise head, no speech and poor social contact, hypotonia

Abbreviations: CZP, clonazepam; d, day; DD, development delay; F, female; L, left; LEV, levetiracetam; M, male; mo, month; MRI, magnetic resonance imaging; NZP, nitrazepam; OXC, oxcarbazepine; P, patient; PB, phenobarbitone; SE, status epilepticus; Sz, seizure; TPM, topiramate; y, year; VPA, valproic acid.

Patient 1 and Patient 2 were siblings. Patient 1 was a 10‐month‐old boy referred to our hospital with seizures and prior developmental delay at the age of 4 months. The seizures were characterized by cyanosis, eye deviation, and focal tonic or clonic seizures lasting approximately 1 minute. His body weight, height, and head circumference at 10 months were 9.0 kg, 74 and, 47 cm, respectively, and all of them were in normal range ($\bar{X}\pm \text{SD}$). He presented poor eye contact, unable to sit independently, or crawl on the referral. His neurologic examination showed remarkable hypotonia and dysmorphic features such as a high forehead, flattened nasal bridge, and hypertelorism. He was delivered by cesarean section at a gestational age of 38 weeks due to maternal pregnancy‐induced hypertension. His parents were healthy and non‐consanguineous. No significant family histories of neurological diseases were documented. The initialized levetiracetam (LEV) reduced the frequency of seizures by around 50% in the first month, but no added benefits for seizure control were observed with dose titration (maximum 45 mg/Kg/d) over time. Subsequently, phenobarbital (5 mg kg^−1^ day^−1^) and oxcarbazepine (maximum 43 mg kg^−1^ day^−1^) were introduced. However, no responses to these antiepileptic drugs (AEDs) were observed and status epilepticus (SE) was predisposed and common. His neurodevelopment regressed following the onset of seizures. He died of SE at the age of 2 years and 3 months; no autopsy was performed.

Patient 2 was the younger sister of Patient 1. She was also delivered by cesarean section at a gestational age of 36^+1^ weeks because of premature placental abruption and was hospitalized on account of neonatal pneumonia. Her physical growth, including head circumference, was in the normal range. She showed neurodevelopmental retardation at 5 months, and seizures started 11 months later. The initial LEV was ineffective for the seizure control and was discontinued subsequently. Seizure freedom was achieved and lasted for more than 2 months following treatment with valproic acid (VPA), nitrazepam (NZP) combined with topiramate (TPM). Similar to her older brother, Patient 2 presented severe developmental regression, refractory seizures, and frequent SE.

Patient 3 was admitted to our hospital at age 4 months because of repeated seizures, and his developmental milestones were within normal range at the onset. The seizures were presented as cyanosis, eye deviation, partial tonic, and clonic seizures with subsequent generalization or not. He had notable hypotonia and subtle dysmorphic features such as a high forehead. His body weight, height, and head circumference were all in normal limits. He was delivered by cesarean section at 31^+6^ weeks of gestation because of premature placental abruption and hospitalized due to preterm infant and low birth weight baby. His parents were healthy Chinese and non‐consanguineous. He had no siblings or significant family history of neurological diseases. The patient was unresponsive to the introduced AEDs comprising LEV, VPA, clonazepam, and TPM, and exhibited obvious neurodevelopmental stagnation. At the last visit aged 1 year 9 months, he was unable to sit independently, had poor eye contact, and no meaningful words. Refractory seizures and frequent SE were also observed.

**Figure 2:**
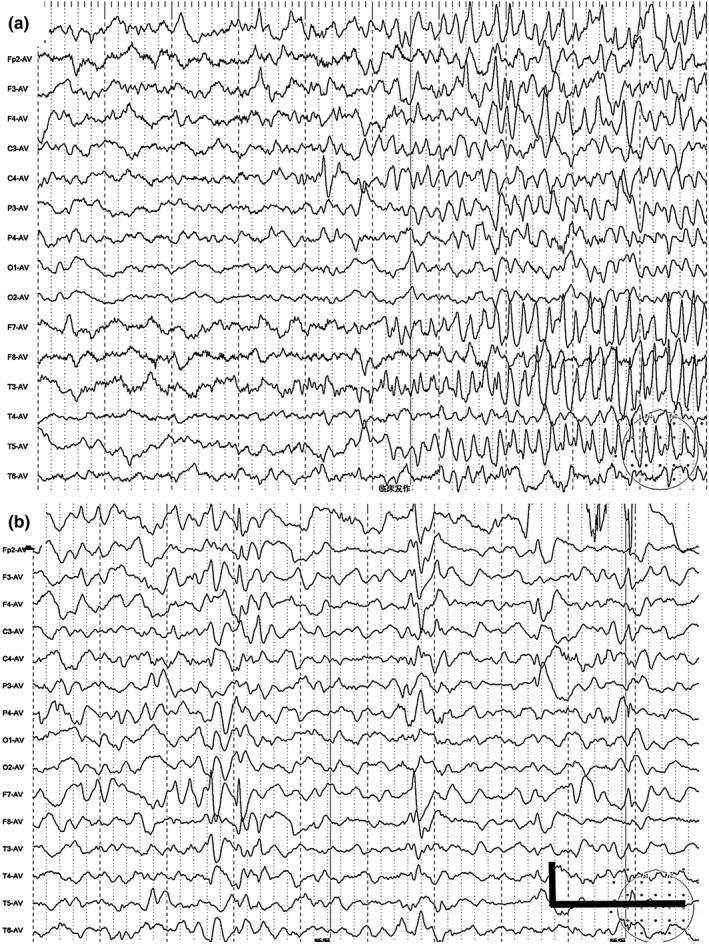
EEGs of subjects carrying SZT2 mutations. Epileptic discharges originating from the left (L) medial temporal area on ictal EEG at 10 months in Patient 1 (a); multi‐focal discharges were recorded at 10 months in Patient 3 (b). Scale bar, 100 μV/s

As shown in Table 1, the initial EEG examinations showed nonspecific alterations in background, without epileptic discharges in the siblings. Patient 3 showed normal interictal EEG recordings, even after the onset of seizures. Over time, ictal epileptic discharges originating from the focal area were recorded in Patient 1 (Figure 2a) and Patient 2. Multiple focal discharges (Figure 2b) on the interictal EEG and slow background were recorded in all the cases, which justified the contribution of epileptic activity to the severe cognitive and behavioral impairments in these cases of DEEs.

**Figure 3:**
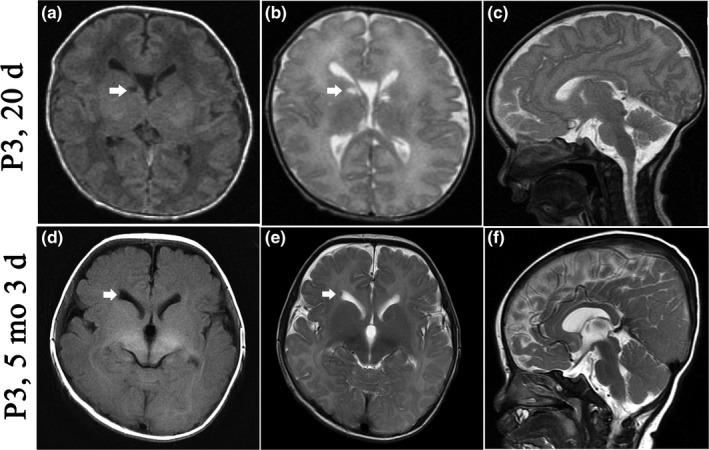
Serial cranial MRI images of Patient 3 with SZT2 mutations. Cystic lesions (white arrow) and widened cavum septum pellucidum were found at 20 day after birth (a, b, c), and the latter was still present at 5 months. Subependymal nodules (white arrow), enlarged ventricle, and shortened corpus callosum were also detected at 5 months (d, e, f)

Regarding the cranial magnetic resonance imaging (MRI), as shown in Figure 3, only Patient 3 presented shortened corpus callosum that has been previously reported in other cases (Pizzino et al., 2018), while Patients 1 and 2 had normal corpus callosum. Transient subependymal cysts and widened cavum septum pellucidum were found in Patient 2. Of note, subependymal nodules (Figure 3), similar to those found in tuberous sclerosis complex (TSC), were presented in Patient 3, while no specific skin changes, such as TSC, were found.

## 4 DISCUSSION

In the present study, four novel compound heterozygous mutations of SZT2 were identified and predicted to be pathogenic due to the truncation mutations (one splice‐site mutation, two frameshift mutations caused by duplication and insertion of a single base‐pair, and one point mutation resulting in a premature stop codon) and the reported related phenotypes. The patients carrying the four novel mutations all presented with epileptic encephalopathies, a phenotype consistent with other previously reported cases (Basel‐Vanagaite et al., 2013; Tsuchida et al., 2018; Venkatesan, Angle, & Millichap, 2016). Compared with the previously reported cases (Basel‐Vanagaite et al., 2013; Tsuchida et al., 2018; Venkatesan et al., 2016), the affected siblings in our study both presented with developmental delay initially, followed by refractory seizures. Therefore, our findings expand the genotypes and phenotypes of SZT2 mutation‐related DEEs. These findings also provide further evidence in support of the concept of DEEs, emphasizing a genetic component in the mechanism of both the developmental delay and epilepsy (Hamdan et al., 2017).

To our knowledge, since the first case reported by Basel et al. (Basel‐Vanagaite et al., 2013) in 2013, only 21 cases (Basel‐Vanagaite et al., 2013; Domingues et al., 2019; Falcone et al., 2013; Imaizumi, Kumakura, Yamamoto‐Shimojima, Ondo, & Yamamoto, 2018; Kariminejad et al., 2018; Nakamura et al., 2018; Naseer et al., 2018; Pizzino et al., 2018; Tsuchida et al., 2018; Venkatesan et al., 2016) (including the three cases presented here) carrying SZT2 mutations have been reported to date. The phenotypes were largely heterogeneous and formed a continuum of characteristics from early‐onset epileptic encephalopathy to mild ID without epilepsy, which may be associated with the alteration in residual protein function because truncating mutations cause complete loss of SZT2 function. Among them, all the cases (18/21, 86%), except three individuals from one family (Falcone et al., 2013), were subsequently diagnosed as epileptic encephalopathies and were refractory to AEDs. In combination, these data suggest that epilepsy and developmental delay are the core symptoms of patients with SZT2 mutations.

In the previously reported cases, corpus callosum abnormalities, comprising atrophy and dysgenesis, were the most common manifestation in cranial MRI (Pizzino et al., 2018). In our study, only Patient 3 was presented with short corpus callosum. In accordance with the previously reported cases, transient cystic lesions, enlarged ventricles, widened cavum septum pellucidum, and delayed myelination were also found (Pizzino et al., 2018). However, the correlation between the specific alterations in cranial MRI and SZT2 mutations is not yet clear.

It has recently been reported that SZT2 dictates GATOR control of mTORC1 signaling under conditions of glucose or amino acid deprivation (Peng et al., 2017). Therefore, it can be speculated that loss‐of‐function SZT2 mutations may lead to the activation of mTORC1 signaling. Dysregulation of this pathway has been implicated in increased cell proliferation and growth, and the subsequent formation of hamartomas, as well as disturbed connectivity of the brain and epileptogenesis affecting ion channel expression and synaptic plasticity (Schubert‐Bast et al., 2018). Subependymal nodules in MRI in P3 as similar as TSC may be related to the activation of mTORC1 signaling secondary to SZT2 mutations.

Collectively, we describe three patients carrying compound heterozygous novel mutations in SZT2 who were presented with DEEs. Of note, one patient exhibited a TSC‐like phenomenon in cranial MRI. Our study indicates that high susceptibility and intractability of SE in DEE, as well as relative specific brain MRI findings suggest potential biallelic mutations of SZT2.

## CONFLICT OF INTEREST

None of the authors have any conflict of interest to declare.

## AUTHOR CONTRIBUTIONS

Xiaomin Sun‐collection of clinical data, patients’ follow‐up, manuscript preparation; Xuefei Zhong‐EEG review, EEG and MRI figure preparation; Tingsong Li‐conception and design, manuscript preparation and critical revision.
